# Disentangling (new) labour market divides: outsiders’ and globalization losers’ socio-economic risks in Europe

**DOI:** 10.1007/s11135-022-01414-9

**Published:** 2022-05-30

**Authors:** Marcello Natili, Fedra Negri

**Affiliations:** Università Degli Studi Di Milano - Bicocca, Milan, Italy

**Keywords:** Labour market outsiders, Globalization losers, Income insecurity, Unemployment risk, Social policies, European countries

## Abstract

In post-industrial and globalized economies, socio-economic risks have become ubiquitous for workers. Two segments of the labour force seem particularly exposed: namely, outsiders (atypical workers and unemployed individuals) and globalization losers (unskilled workers in offshorable employment sectors), with relevant consequences for party competition in Europe. The coexistence of these two segments of vulnerable workers has brought conceptual ambiguity. Using the original 2019 REScEU Mass Survey on ten European countries, we firstly clarify that outsiders and globalization losers do not constitute the same socio-economic group. Secondly, we look into the micro-foundations of outsiders’ and globalization losers’ redistributive preferences and political behaviours by showing that outsiderness, rather than exposure to international competition, constitutes a significant driver of income and employment insecurities, and of dependency on social protection and family financial aid.

**Supplementary Information:**

The online version contains supplementary material available at 10.1007/s11135-022-01414-9.

## Introduction

Over the last decades, European labour markets have become increasingly fragmented and unequal. Thus, scholars in political economy, comparative politics and social policy analysis have put effort into theorizing the structures and investigating the socio-economic and political consequences of two new well-entrenched labour market divides. Firstly, the shift to a post-industrial economy and the related flexibilization of the labour market have created the potential for conflict among workers, the so-called insider–outsider divide (e.g. Emmenegger et al. 2012; Marx 2014; Marx & Picot 2013; Rovny & Rovny 2017; Rueda 2005; Schwander & Häusermann 2013; Vlandas 2020). Secondly, increasing global competition and the possibility of outsourcing production in developing economies have fostered a divide between the so-called winners and losers of globalization (e.g. Dancygier & Walter 2015; Kriesi et al. 2006; Teney et al. 2014; Walter 2010, 2017; Wren & Rehm 2013). Automation and technological change have further exacerbated such dynamics. Indeed, on the one side, the gig economy has contributed to the rapid diffusion of so-called platform workers (Rahman & Thelen 2019); on the other, new technologies have caused work that was traditionally sheltered from international competition suddenly to be more exposed (Autor et al. 2003; Kaihovaara & Im 2020; Rommel & Walter 2018).

Given these labour market transformations and the related divides, a lively debate exists among scholars regarding if, and how, such divides have the potential to transform politics (e.g. Häusermann et al. 2020; Kriesi et al. 2006; Langsæther & Stubager 2019; Marx 2014; Negri 2019; Rommel & Walter 2018; Rovny & Rovny 2017). These works generally share the assumption that vulnerable workers are particularly exposed to socio-economic risks, which, in turn, shape first their desired degree of redistribution and state intervention in the economic sphere, and then their voting behaviours and party preferences. However, as outlined by Rehm (2009, p. 856), “although many macro theories explicitly or implicitly rely on them, these individual-level mechanisms are usually only stated as assumptions and remain largely untested”.

Against this background, our study advances the existing scholarship both analytically and empirically. We build on the latest analytical contributions (Häusermann et al. 2020; Marx & Picot 2020; Vlandas 2020; Rommel & Walter 2018) and exploit the rich information provided by the original Reconciling Economic and Social Europe: Values, Ideas and Politics (REScEU) Mass Survey (Donati et al. 2021) to disentangle common properties and differences among outsiders (i.e. atypical workers and unemployed individuals) and globalization losers (i.e. low-skilled workers in sectors exposed to global competition). We maintain that properly unpacking the socio-economic risks to which these two segments of vulnerable workers are exposed is crucial to understand how these new labour market divides affect party politics and social policymaking in established democracies. Indeed, a lack of clarity puts researchers at risk of conflating all workers in vulnerable positions in the same group, assuming they share similar policy and political preferences, and confusing the effects of labour market transformations with those of other possible determinants of political behaviour. Moreover, a more precise characterization of new labour market divides allows us to speculate on which are the best policy solutions to address the related socio-economic insecurities—whether to re-regulate the labour market to curb labour market segmentation and/or address the rules on outsourcing and de-localization of companies and production sites.

Our results provide novel empirical support for Häusermann’s (2020) original argument that globalization losers and outsiders are conceptually and empirically separate groups. Tabular analysis clarifies that unemployment and atypical employment do not affect individuals working in offshorable sectors more than those working in sheltered sectors.

Secondly, and more relevantly, our study enhances theoretical and empirical knowledge about the micro-foundations of outsiders’ and globalization losers’ redistributive preferences and voting behaviours by shedding light on the socio-economic risks to which they are exposed. Indeed, though there are a few empirical works testing the exposure of one of these two segments of vulnerable workers to a specific risk (e.g. Burgoon & Dekker 2010; Häusermann et al. 2015; Kaihovaara & Im 2020; Schwander & Häusermann 2013; Walter 2017), no study, to our knowledge, has systematically compared the risk exposure of outsiders and globalization losers. Furthermore, original questions included only in the REScEU Mass Survey allow us to introduce a new multi-dimensional conceptualization of risk exposure that goes beyond employment insecurity to focus also on income insecurity, access to social protection, and dependency on family financial assistance. This multi-dimensional conceptualization of risk exposure allows us to have a more precise understanding of the specific vulnerabilities and needs of these new and, apparently, particularly vulnerable segments of the labour force. Our results show that rather than exposure to international competition, unemployment and atypical employment constitute significant drivers of our multi-dimensional conceptualization of risk exposure, thus providing fresh insights into the partially competing micro-logics of different strands of literature on new labour market divides and their consequences for party politics (Häusermann et al. 2020).

The article is organized as follows. Section 2 reviews the literature on labour market transformations, and details the expectations and the analytical goals that ground our analysis. Section 3 introduces the dataset, clarifies the operationalization of the variables, and details the model specifications. Sections 4 and 5 host the analysis. In Sect. 6, we summarize our findings, discuss their implications for the literature on the structuring of new political conflicts in Europe, and suggest directions for future research.

## New divides and risks in post-industrial and globalized economies: theoretical underpinnings and pitfalls

Workers in post-industrial and globalized economies can hardly be considered as a homogeneous class facing similar risks. Indeed, since the late 1970s, governments have encouraged labour market deregulation to enhance the job creation potential of the service sector, thereby resulting in the proliferation of part-time, atypical and temporary employment contracts to the detriment of standard (i.e. full-time and open-ended) ones. The presence of workers hired under different contracts, each with different access to legal and social protection, has divided labour into segments of more sheltered and more vulnerable workers, a process known as the dualization of the labour market (Emmenegger et al. 2012). Though a lively debate on the operationalization of this dualism exists (e.g. Marx & Picot 2020; Rovny & Rovny 2017; Schwander & Häusermann 2013; Vlandas 2020), the original formulation of the insider–outsider theory, mainly developed in political economy, maintains that distinct legal categories of employment contracts unevenly distribute risk among workers, thus differentiating between ‘insiders’ and ‘outsiders’. Insiders are workers hired under open-ended contracts, while outsiders comprise both workers hired under atypical or temporary contracts and unemployed people actively searching for jobs, who find it hardest to endure economic fluctuations (Rueda 2005).

Alongside labour market deregulation, a flourishing strand of literature, mainly in comparative politics, has pointed to the process of globalization as a prominent source of new insecurities and risks for workers (Kriesi et al. 2006; Teney et al. 2014), even though its effects are unevenly distributed across employment sectors and job tasks (Scheve & Slaughter 2004; Walter 2010, 2017; Wren & Rehm 2013). On the one hand, globalization of trade and production in sectors exposed to international competition poses a substantial threat to workers whose jobs can theoretically be performed abroad. On the other hand, many jobs simply cannot be offshored because the services they provide require them to be on-site (Blinder 2009), and so many workers remain almost unaffected by global competition (Dancygier & Walter 2015; Rommel & Walter 2018). Globalization thus fosters a divide between workers employed in non-offshorable sectors and occupations, which are sheltered, and workers at risk of losing their jobs as they may be offshored to domestic workers abroad. However, this is not the end of the story, as globalization may also constitute an opportunity for specific segments of the labour force. Indeed, highly educated individuals in internationally competitive industries and occupations may improve their labour market positions by being able to sell their services, skills and competences in a global, and therefore wider, labour market.

To capture the divide among workers determined by globalization, scholars have suggested considering two factors: firstly, whether individuals work in sectors exposed to international competition; and, secondly, their skill level (Dancygier & Walter 2015; Owen & Johnston 2017; Rommel & Walter 2018). Unskilled individuals working in sectors open to international competition are the globalization losers, while skilled individuals working in the same sectors are the globalization winners*.* As to those working in sectors that are sheltered from international competition, the difference between unskilled and skilled workers should be less pronounced, as unskilled workers should be less exposed to socio-economic risks compared to their counterparts in offshorable sectors, while skilled workers should enjoy less favourable working conditions than the winners, particularly in terms of remuneration (Walter 2017).

In light of this theoretical background, we identify three main grey areas deserving more fine-grained research to disentangle whether and to what extent labour market deregulation and the process of globalization translate into socio-economic risks and vulnerabilities tangibly suffered by individuals in their everyday lives.

Firstly, both the insider–outsider and the globalization winners-losers strands of literature signal the existence of vulnerable segments of the labour force, facing higher socio-economic risks than the rest of the working population. Though these two theoretical accounts have evolved almost entirely separately, have different theoretical priors and outline different mechanisms (i.e. deregulation vs globalization), such a common starting point has facilitated conceptual ambiguity. Indeed, the (few) studies that connect the two processes implicitly or explicitly tend to consider globalization losers as roughly congruent with outsiders (e.g. Lubbers et al. 2002; King & Rueda 2008), a thesis that is explicitly contested by Häusermann (2020, p. 381). Moreover, it is unclear whether the offshorability risk on the one hand, and the atypical employment and unemployment risks on the other hand, are associated, so that being exposed to increasing economic competition increases the probability of being hired under atypical or temporary contracts or of being unemployed.

Secondly, there is considerable theoretical and empirical ambiguity concerning which socio-economic risks these two segments of vulnerable workers face in today’s labour markets. Most of the insider–outsider literature, and especially the strand pertaining to legal employment contracts, focuses on a single risk: atypical employment and unemployment generate uncertainty about future income (Marx 2014, p. 138; see also Rueda 2005). Yet individual perceptions of insecurity most likely depend heavily on present income and purchasing power. This aspect is indeed acknowledged by scholars investigating the risk exposure of globalization losers, who are described as particularly affected by both the risk of unemployment and low wages (Walter 2010, 2017).

Moreover, the individual’s present and future income may depend on several institutions: namely, the market, the state, and/or the family (Esping-Andersen 1999). If the market sphere is always taken into consideration—though not always focusing on both income and employment insecurities—less attention is normally paid to the latter two institutions.

Starting from the state, the institutional architecture of the welfare system may reduce, or even reinforce, existing labour market divides. Indeed, specific categories of workers may have difficulties gaining access to social benefits because of their incomplete contribution records, or to wage supplementation schemes because of discriminating sector-based rules on entitlement conditions. Similarly, non-contributory needs-based social programmes have very heterogeneous degrees of inclusiveness, so that only individuals in extreme poverty can access such programmes in some cases, whereas, in others, the programmes are much more inclusive and also protect vulnerable workers (Natili 2019).^1^ In other words, some workers may be institutionally excluded from one or even all types of social benefit, and so the welfare state itself may constitute a source of socio-economic risk—the so-called social protection dualism (Schwander & Häusermann 2013).

Moving to the family, this institution is important in mitigating market insecurities and providing economic support, particularly where state public support is less inclusive and generous. Indeed, it is well known that in some European countries, traditionally in Southern Europe, families have the main task of caring for their members and are ultimately responsible for their wellbeing because of the principle of subsidiarity (Ferrera 1996). However, depending on the family may limit individual autonomy and mobility, thus further accentuating disadvantages in the labour market. Moreover, not all families are able to provide financial help, and the capability of families from different socio-economic backgrounds to provide adequate support is highly heterogeneous. In other words, depending on family help, especially for some individuals, can also be a significant source of vulnerability and risk.

To disentangle precisely who outsiders and globalization losers are and unpack along different dimensions which socio-economic risks and vulnerabilities they respectively face is relevant, not least because it can shed light on the individual-level mechanisms grounding their redistributive preferences and voting behaviours, which is needed to understand whether outsiders and globalization losers could constitute the core constituencies of similar or different parties. To achieve this goal, we focus not only on income and employment insecurities, but also on the possibility of accessing social protection and financial dependency through the family.

Thirdly, and finally, there is a certain ambiguity concerning the internal consistency of these classifications, in particular regarding the insider–outsider divide. Indeed, if it is pretty straightforward who are the insiders, outsiders usually includes workers with different contract typologies, such as part-time or temporary workers, casual and irregular workers, and the unemployed (Emmenegger 2009). In a different direction, Jansen (2016) has outlined that self-employed individuals without employees (i.e. solo self-employed) are exposed to risks comparable to those suffered by outsiders. It therefore seems worth investigating whether outsiders constitute a coherent group in terms of socio-economic risks, and if the solo self-employed should be included in this group. As to globalization losers, trade today takes place in a world of fragmented production chains which cross-cut industrial sectors (Kaihovaara & Im 2020). In the EU, the international organization of production has increased markedly with Eastern enlargement, as the higher income countries have offshored parts of their production activities to Central and Eastern European countries. It is therefore important to investigate how the risk exposure endured by unskilled workers in offshorable sectors varies between core and Eastern EU countries, given their different positions in the global production chains and welfare regimes.

## Data description and empirical strategy

### The 2019 REScEU mass survey

The empirical analysis adopts a European-wide perspective by taking advantage of the second wave^2^ of the REScEU Mass Survey, an original public opinion survey that covers ten European countries: Finland, France, Germany, Greece, Hungary, Italy, the Netherlands, Poland, Spain and Sweden. The fieldwork was conducted through a CAWI method by IPSOS between June and August 2019 on a sample of about 1,500 respondents aged between 18 and 70 voluntarily registered to the company online panel in each country. The sample has been built through a quota sampling data around gender; age (three categories: 18–34, 35–54, 65+); educational level (based on ISCED2011 categories recoded into: ‘Less than primary, primary and lower secondary education’ for ISCED2011 levels 0–2; ‘Upper secondary and post-secondary non-tertiary education’ for levels 3–4; and ‘Tertiary education’ for levels 5–8); and geographic area of residence. Sampling quotas around gender and age have been defined in each country in proportion to the population data provided by Eurostat 2018. Those around educational levels are based on Eurostat data on population by educational attainment level, sex, age and labour status [edat_lfs_9904]. Finally, with regard to areas of residence, sampling has been carried out by refining NUTS1 categories to guarantee the inclusive sampling of big cities, suburbs and rural areas (Donati et al. 2021).

Given our theoretical focus, the original sample has been restricted to the economically active population, which includes both employed (employees and self-employed) and unemployed people actively searching for a job, but not the inactive population—namely, pensioners, students, housekeepers, those affected by permanent disability, and those in military or community service. Thus, our resulting sample includes 8,085 respondents.

### Variable description and model specification

The empirical analysis develops in two steps. Firstly, Sects. 4.1 and 4.2 present descriptive tabular analysis to show how insiders and outsiders and globalization winners and losers, respectively, are distributed in the full sample and across countries to compare the incidence of these two segments of vulnerable workers in European economies. Section 4.3 then, through the Pearson’s χ^2^ test for tabular association between categorical variables, assesses whether and to what extent an association between being an outsider and being a globalization loser exists in our sample, and whether it is likely to reflect a real association in the population.

Secondly, we investigate whether and how labour market deregulation and the process of globalization expose the affected individuals to special socio-economic risks by estimating four ordinal logit models (Models 1–4) on as many ordinal dependent variables. In line with our theoretical framework, we use four dependent variables to explore different facets of socio-economic risk: namely, income insecurity, employment insecurity, access to social protection and family dependency.

Income insecurity asks respondents about their feelings about their present household income. Answers range from 1 (living comfortably on present income) to 4 (finding it very difficult on present income).

Employment insecurity asks respondents whether, in the last two years, they have experienced a continuous period of unemployment. Answers go from 1 (never unemployed) to 4 (yes, unemployed for more than 12 months). This dependent variable allows us to cast a light on whether respondents experienced being unemployed in the last two years and on the duration of their unemployment spells, thus discriminating between long- and short-term unemployment. In other words, it allows us to test to what extent different categories of worker are exposed to different forms of unemployment.

Access to social protection investigates whether respondents have received one or more types of social benefit in the last two years, excluding old age/survivor pensions (e.g. unemployment benefit, family transfer, housing allowance, minimum income scheme, sickness or disability benefit), and whether these benefits constitute the main or a very important source of their household income. Answers are 1 (no), 2 (yes, but they are not the main source of income), and 3 (yes, and they are the main source of income.)

Lastly, family dependency asks respondents how often they get financial help from close family or friends to pay bills, mortgage or rent, school fees or medical expenses. Answers range from 1 (never) to 4 (often).

Accordingly, these four ordinal dependent variables are four-point scales, with the exception of access to social protection, which is a three-point scale.

Models 1–4 include the same independent variables of interest to capture the status of outsider and that of globalization loser. The categorical variable ‘labour market status’ distinguishes among employers, solo self-employed, insiders (i.e. employees with open-ended contracts – reference category), atypical workers and unemployed. In this setting, we mainly focus on unemployed and atypical workers, as they are undoubtedly treated as outsiders in the literature (Emmenegger 2009; Marx 2014; Rueda 2005). However, we look also at solo self-employed, as a contribution suggested that they might be exposed to risks comparable to those faced by the unemployed and atypical workers (Jansen 2016). Though this operationalization only grasps a static, contractual-based conception of outsiderness, alternative indicators (e.g. Rehm 2009; Schwander & Häusermann 2013) are not suitable for our study as they use individuals’ occupational categories and skill level to predict their labour market risk. However, such individual characteristics are also classic predictors of the status of globalization loser, our second variable of interest. Moreover, such alternative indicators account for age and gender, which are classic predictors of socio-economic risks and vulnerabilities on their own (for a systematic comparison, see Rovny & Rovny 2017).

Moving to the status of globalization loser, the dummy variable ‘offshorable’ is equal to 1 if respondents work in offshorable sectors and 0 if they work in sheltered sectors. To classify sectors as offshorable or not, we match respondents’ occupation as provided by the REScEU Mass Survey with information about the potential for offshoring of each occupational category (i.e. ISCO codes) as provided by Rommel and Walter (2018), which, in turn, is grounded on the offshorability index developed by Blinder (2009). Moreover, given that the literature maintains that the offshorability risk individuals face is also due to their skill level, offshorable is interacted with the categorical variable ‘skill level’, which allows us to distinguish among low- (ISCED2011 levels 0–2), medium- (levels 3–4), and high-skilled (levels 5–8) workers.

Conventional control variables are included in Models 1–4 (see Appendix Table 3): age (reference category: 18–34), gender (reference category: male), having a partner, having children, living in urban areas, part-time work and trade union membership. Country dummies control for idiosyncratic country characteristics (reference country: Sweden). Finally, in Models 5–8 (see Appendix Table 4), we replicate the same analysis by substituting country dummies with the categorical variable ‘welfare regime’ that distinguishes among Southern (Greece, Italy and Spain), Eastern (Hungary and Poland – reference category), Continental (France, Germany, and the Netherlands), and Nordic (Finland and Sweden) countries. ‘Welfare regime’ enters Models 5–8 in interaction with ‘offshorable’. For further details, Appendix Table 1 lists the variables’ names and operationalization and links each variable with the corresponding item in the REScEU Mass Survey. Appendix Table 2 provides descriptive statistics. Appendix Tables 3 and 4 report the full model specifications and the coefficient estimates.

## Outsiders and globalization losers in the REScEU mass survey

### The insider–outsider divide

**Fig. 1:**
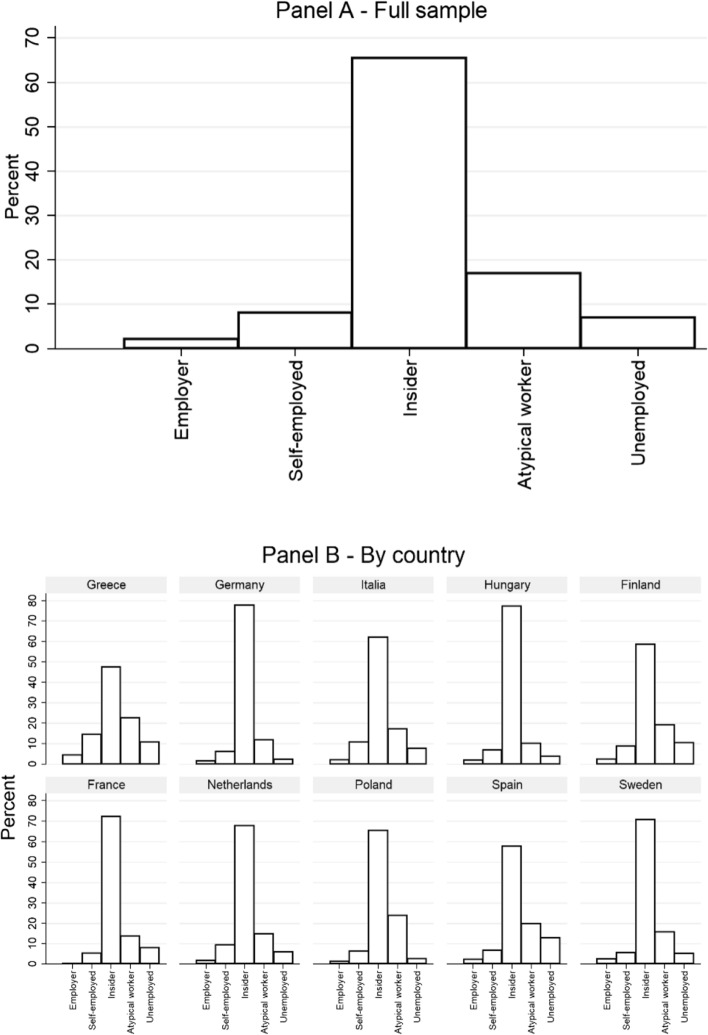
Distribution of insiders and outsiders

Figure 1 panel A displays the distribution of insiders, outsiders, solo self-employed and employers in the REScEU Mass Survey. Not surprisingly, insiders are the most represented group in our sample, being 66 per cent of the active labour force, followed by outsiders (24%), who can be usefully divided into workers with fixed-term and atypical contracts (17%) and unemployed (7%). Employers account for 2 per cent, while solo self-employed are almost 8 per cent of the active labour force.

Cross-country comparisons shown in Fig. 1 Panel B provide some interesting information.^3^ Insiders constitute a share of the workforce larger than the sample mean in Continental, Eastern and Nordic countries (i.e. France 72%, Germany 78%, Hungary 77% and Sweden 71%), while insiders account for a share of the workforce smaller than the sample mean in Southern European countries (i.e. Greece 48%, Italy 62% and Spain 58%). Finland (59%) is the exception to this general rule.

Almost symmetrically, the share of outsiders is lower than the sample mean in Continental, Eastern and Nordic countries, while it is particularly high in Southern Europe (for similar results, see Prosser 2016). In detail, the share of employees with fixed-term and atypical contracts is lower than the sample mean in Germany (12%), Hungary (10%) and France (14%), while it is particularly high in Greece (23%), Spain (20%) and Poland (24%). Moving to the unemployed, their share is lower than the sample mean in Sweden (5%), Germany (2%), Hungary (4%) and Poland (3%), while it is higher than the sample mean in Greece (11%), Spain (13%) and Finland (11%). Relevantly, both patterns are largely consistent with labour market statistics provided online by Eurostat (year of reference: 2019).

The different weights of the insider–outsider divide within European labour markets become particularly evident if we consider solo self-employed as a potential new entrant category in the outsider world, as their share is above average in Southern Europe (see also OECD 2022), particularly in Greece (14%) and Italy (11%), while it is below average in France (5%), Germany (6%), Hungary (7%), Poland (6%) and Sweden (5%).

### The globalization winner-loser divide

**Fig. 2:**
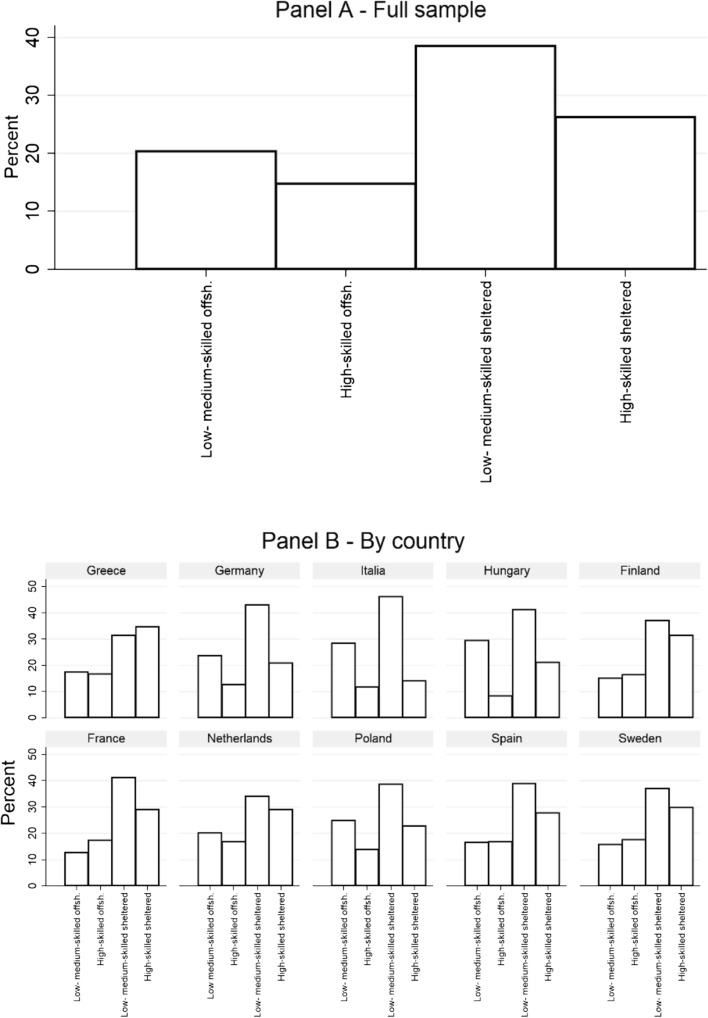
Distribution of globalization winners and losers

As displayed in Fig. 2 Panel A, the most represented group in the REScEU Mass Survey corresponds to individuals working in sheltered sectors (65%), which can be usefully divided into low- and medium- (39%)^4^ and high-skilled (26%). Focusing on offshorable sectors, both globalization losers (low- and medium-skilled, 20%) and winners (high-skilled, 15%) constitute a relevant share of the workforce in our sample. Importantly, this distribution is consistent with most of the literature in the field, including Blinder (2009) and Dancygier and Walter (2015).

Here, too, cross-country comparisons provide interesting information. In detail, the share of globalization losers is above average in Germany (24%), Hungary (29%), Italy (28%) and Poland (25%), while it is below average in France (13%), Finland (15%), Greece (17%), Spain (17%) and Sweden (16%). Globalization winners account for a share of the workforce higher than the sample mean in France (17%) and Sweden (17%), while Germany (13%), Italy (12%) and Hungary (8%) display a share of globalization winners lower than the sample mean. The higher presence of globalization losers in predominantly manufacturing countries—Germany, Italy, Hungary and Poland—is consistent with Blinder (2009).

### Disentangling labour market outsiders and globalization losers

Having described how outsiders and globalization losers are distributed in the full sample and across countries, we assess whether and to what extent an association between these two segments of vulnerable workers exists. Table 1 accomplishes this goal by displaying the distribution of labour market statuses by workers’ skill levels and sectors’ exposure to international competition, and by computing the Pearson’s χ^2^ test for tabular association between categorical variables. Table 1 also provides the observed frequencies, the expected frequencies in case of no association, and the contribution of each cell to the Pearson’s χ^2^ statistic. Note that, to assign currently unemployed individuals to offshorable or sheltered sectors, we refer to their last occupation.

**Table 1:** Outsiders’ distribution by skill level and offshorability

	Offshorable sector		Sheltered sector		Full sample
	Low/medium skilled Globalization losers	High skilled Globalization winners	Low/medium skilled	High skilled	
Unemployed*					
*Outsiders*					
Frequency	141	54	273	104	572
Exp. frequency	116.7	84.6	220.5	150.3	572.0
*χ*^*2*^* contribution*	5.1	11.1	12.5	14.2	42.9
***Column %***	**8.55**	**4.52**	**8.76**	**4.90**	**7.07**
**Atypical worker**					
*Outsiders*					
Frequency	254	173	589	363	1,379
Exp. frequency	281.3	204.0	531.5	362.3	1,379.0
χ^2^ contribution	2.6	4.7	6.2	0.0	13.6
***Column %***	**15.40**	**14.46**	**18.90**	**17.09**	**17.06**
**Self-employed**					
*Potential new entrant category*					
Frequency	128	144	238	150	660
Exp. frequency	134.6	97.6	254.4	173.4	660.0
χ^2^ contribution	0.3	22.0	1.1	3.2	26.6
***Column %***	**7.76**	**12.04**	**7.64**	**7.06**	**8.16**
**Insider**					
Frequency	1,101	800	1,951	1,445	5,297
Exp. frequency	1,080.4	783.6	2,041.5	1,391.6	5,297.0
χ^2^ contribution	0.4	0.3	4.0	2.1	6.8
***Column %***	**66.77**	**66.89**	**62.61**	**68.03**	**65.52**
**Employer**					
Frequency	25	25	65	62	177
Exp. frequency	36.1	26.2	68.2	46.5	177.0
χ^2^ contribution	3.4	0.1	0.2	5.2	8.8
***Column %***	**1.52**	**2.09**	**2.09**	**2.92**	**2.19**

*To assign currently unemployed individuals to offshorable or sheltered sectors, we refer to their last occupation

Table 1 provides two main messages. Firstly, it shows that almost one third of the individuals that are currently unemployed previously worked in offshorable sectors, two-thirds in sheltered sectors. In more detail, while in the full sample the unemployment risk is 7% (last column), low- and medium-skilled individuals in offshorable sectors are markedly more affected (9%) than their more skilled colleagues (5%). While at first glance this evidence seems to suggest that the unemployment risk is more pronounced among globalization losers, if we look at sectors not affected by international competition we find exactly the same pattern, with the unemployment risk suffered by less skilled individuals (9%) being almost double that suffered by highly skilled ones (5%). Overall, this evidence suggests that the likelihood of becoming unemployed is related more to workers’ skill level (the lower the skill level, the higher the risk) than to the exposure of a given sector to international competition.

Secondly, and even more significantly, Table 1 adds that, irrespective of their skill level, individuals working in sheltered sectors are more likely to be hired under atypical contracts than those working in offshorable sectors. In detail, 19% of low- and medium-skilled individuals working in sheltered sectors have atypical contracts, while this share decreases to 15% for individuals with the same skill level but working in offshorable sectors. Among the highly skilled, 17% of those working in sheltered sectors have atypical contracts, compared with 14% of those working in offshorable sectors. This evidence suggests that atypical employment more heavily affects sectors that are sheltered from international competition and builds a bridge to a recent contribution claiming that low-skilled workers providing non-offshorable essential services (e.g. personal care, cleaning, delivery, catering, transport services, etc.) constitute one of the most insecure segments in today’s labour markets (Palier, 2020).

Broadly speaking, Table 1 shows that unemployment hits low- and medium-skilled individuals more heavily irrespective of the exposure of the sector in which they work to international competition, and that atypical employment affects slightly more workers in sheltered than in offshorable sectors.

However, this is not enough to argue that there is no association between our two categorical variables. Indeed, the Pearson’s χ^2^ statistic (98.6445; *p*-value = 0.000) on the full sample confirms that an association exists. In this case, the contribution of each cell to the Pearson’s χ^2^ statistic is highly informative: indeed, it clarifies that such an association is not given by low-skilled unemployed or atypical workers working in offshorable sectors, as we would have expected if there were a relationship between the status of outsider and globalization loser; rather, it is mainly given by unemployed individuals working in sheltered sectors and by highly skilled self-employed individuals working in offshorable sectors. Therefore, such an association does not relate the status of outsider to that of globalization loser. All in all, this evidence lends further support to Häusermann (2020), who firstly underlined how the assumption according to which outsiders coincide with globalization losers is largely mistaken.

## Who risks what? Unpacking the socio-economic vulnerabilities of labour market outsiders and globalization losers

This section investigates the extent to which labour market deregulation and the process of globalization are related to socio-economic vulnerabilities. Crudely put, are outsiders and globalization losers exposed to similar socio-economic risks?

To answer this question, we embrace a multi-dimensional conceptualization of risk exposure by estimating eight ordinal logit models (i.e. four with country dummies and four with ‘welfare regime’) on the dependent variables income insecurity, employment insecurity, access to social protection, and family dependency, each of them pointing to a special vulnerability potentially suffered by respondents due to their contractual form or to the exposure of their employment sector to international competition.

**Fig. 3:**
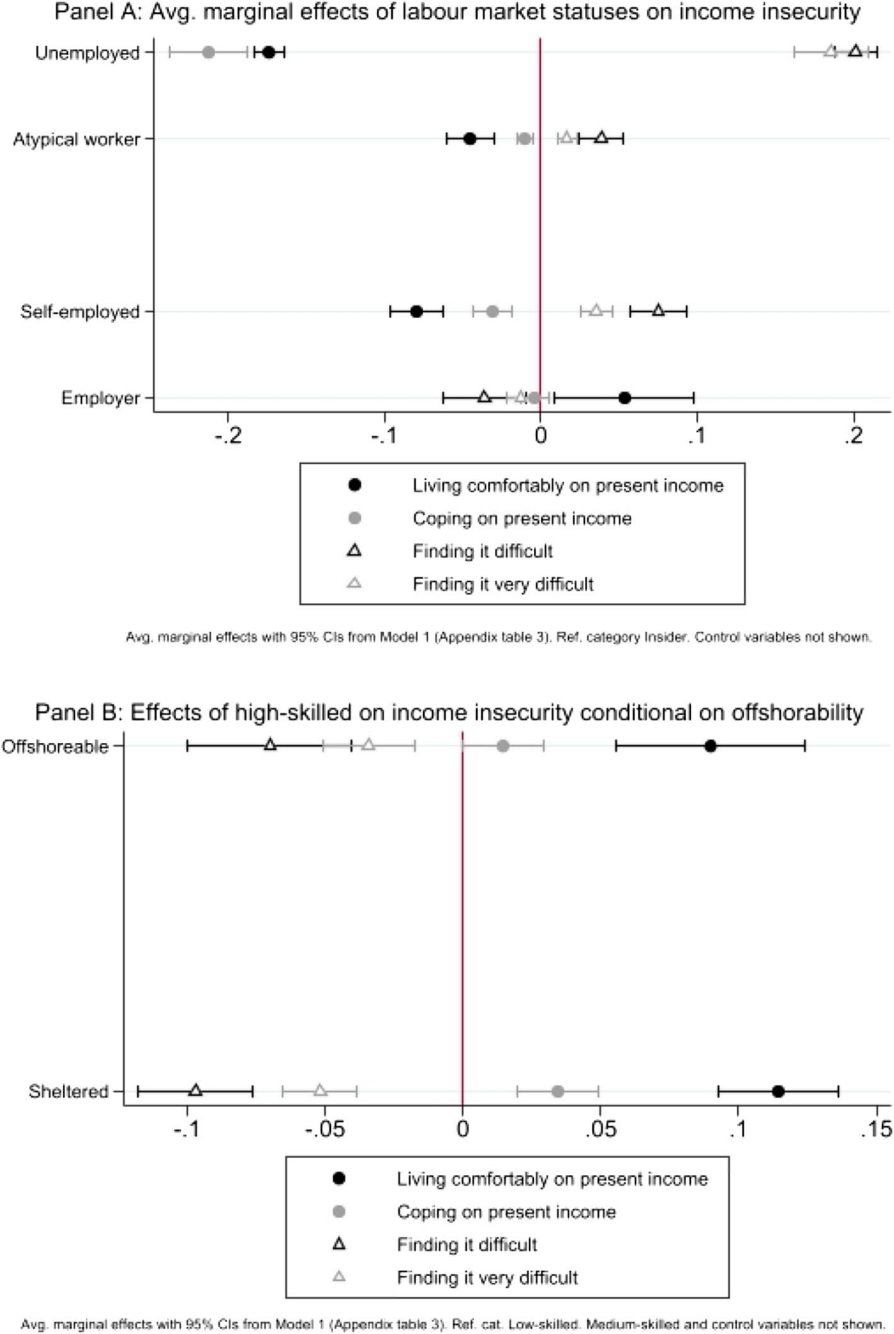
The effects of labour market status and skill level on income insecurity

**Fig. 4:**
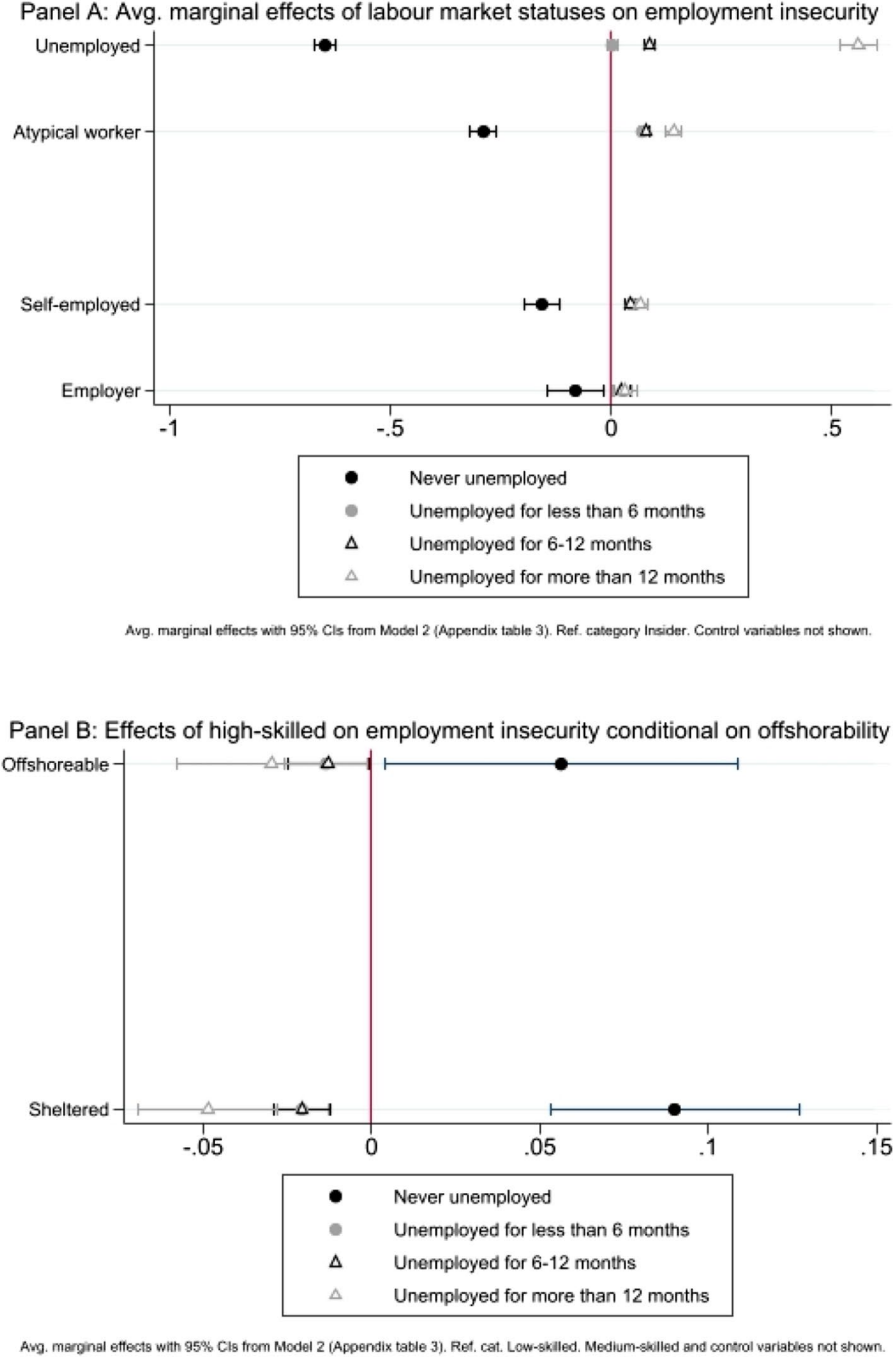
The effects of labour market status and skill level on employment insecurity

**Fig. 5:**
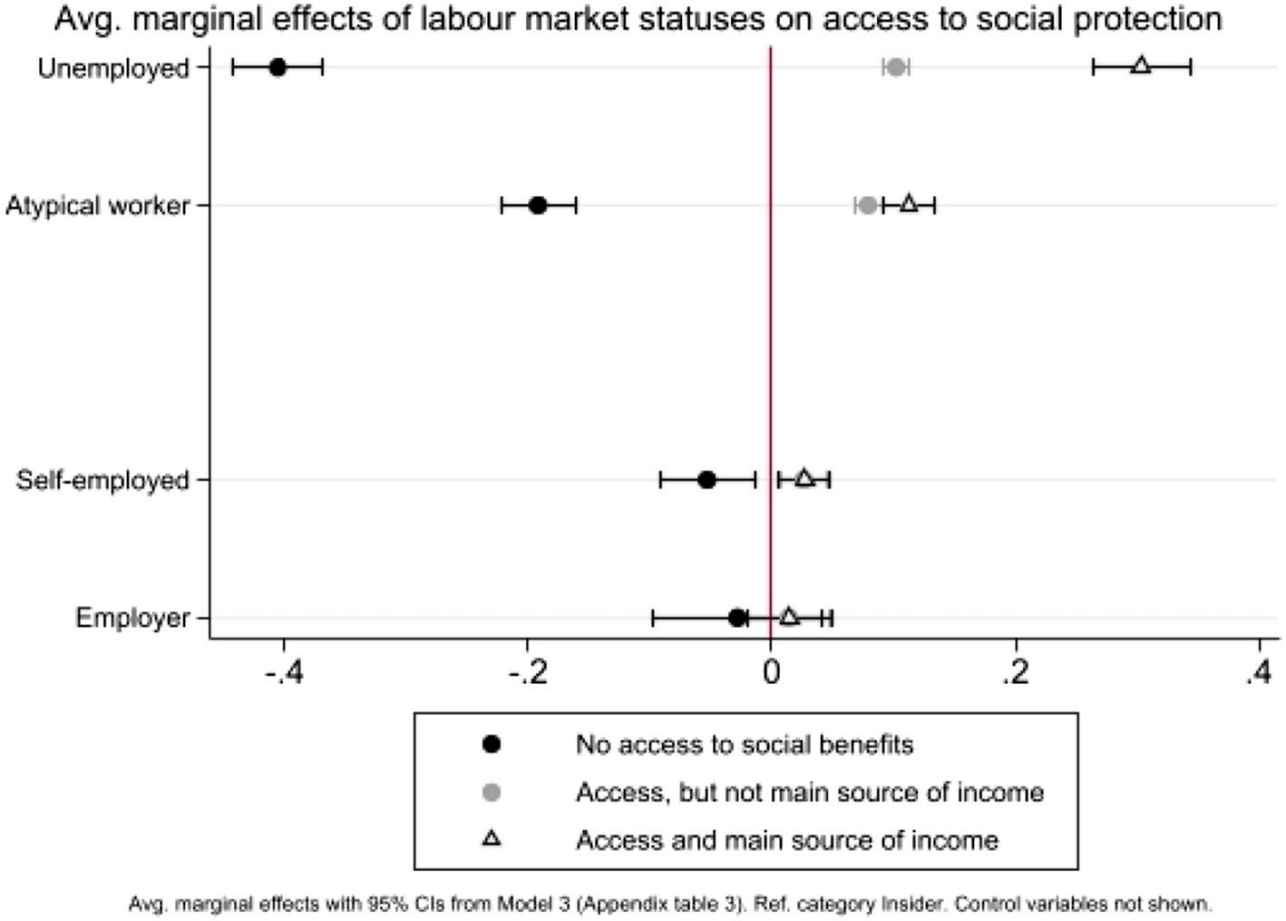
The effect of labour market status on access to social protection

**Fig. 6:**
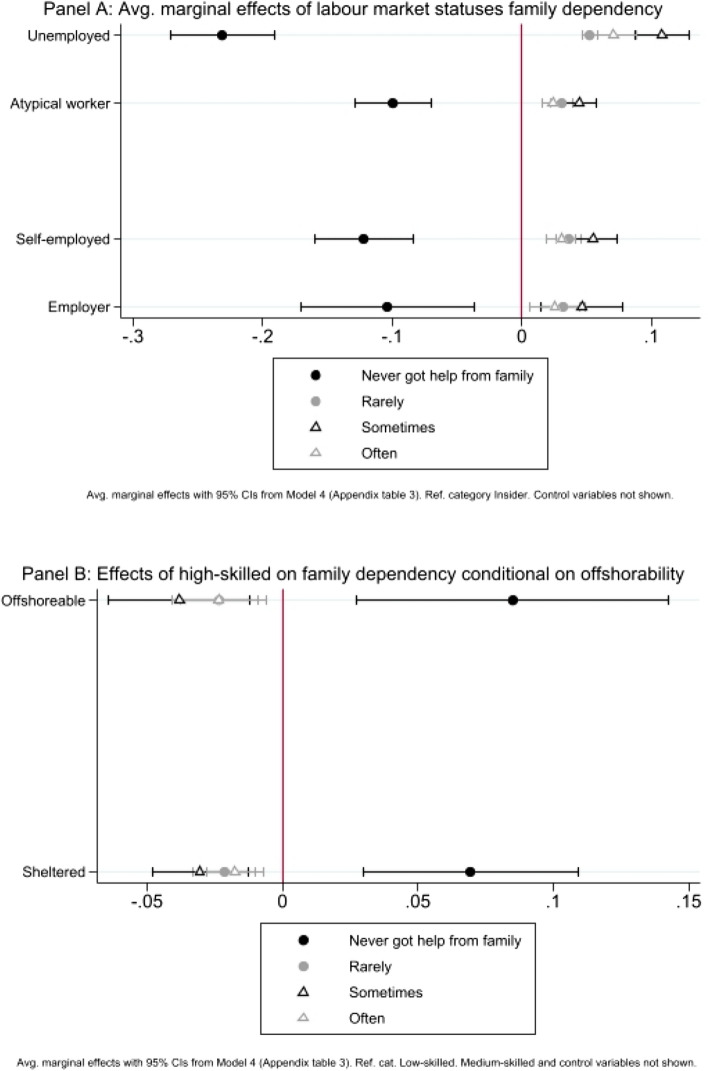
The effects of labour market status and skill level on family dependency

Appendix Tables 3 and 4 report the full model specifications and the coefficient estimates. As ordinal logit coefficients do not carry any substantive meaning beyond their statistical significance, to grasp the direction and magnitude of the associations of interest, Figs. 3, 4, 5, 6 display the average marginal effects of a unitary increase in each independent variable of interest on the probability of choosing a given category of the dependent variable.

### Income insecurity

In Europe, a relevant share of the workforce lives on wages that do not allow them to make a decent living, a phenomenon also known as in-work poverty (Marx & Noland, 2014). But which segments of the labour force? Model 1 (see Appendix Table 3) shows that outsiders are significantly more exposed to in-work poverty than insiders. The average marginal effects in Fig. 3 Panel A suggest that being an atypical worker (versus being an insider) decreases the predicted probability of living comfortably ( − 0.05 points) and coping ( − 0.01) on present income, while it increases the predicted possibility of finding it difficult (+ 0.04) and very difficult (+0.02). The pattern is the same, but with a stronger magnitude, for the unemployed: they are less likely both to live comfortably ( − 0.17) and to be coping (− 0.21) on present income and are more likely to find it hard (+ 0.20 points) than insiders. It is worth noticing that the solo self-employed seem to share with outsiders the burden of income insecurity, though occupying an intermediate position between unemployed people and atypical workers.

If we look at sectors’ offshorability, Model 1 shows that working in occupational sectors exposed to international competition (versus working in sheltered sectors) is unrelated to income insecurity. Indeed, the coefficient of the variable ‘offshorable’ does not reach statistical significance either alone or in interaction with respondents’ skill levels. This means that, once the contractual forms and socio-demographic characteristics are controlled for, the exposure of a given employment sector to international competition does not exercise an autonomous effect on the respondents’ predicted probability of facing income insecurity.

Our results also suggest that skill level autonomously affects income insecurity. As shown by the conditional average marginal effects in Fig. 3 Panel B, highly skilled respondents working in both sheltered and offshorable sectors are more likely to live comfortably (+ 0.11 for sheltered and + 0.09 for offshorable) and to cope (+ 0.03 for sheltered and + 0.01 for offshorable) on present income and are less likely to find it difficult (− 0.10 for sheltered and  − 0.07 for offshorable) or very difficult (-0.05 for sheltered and − 0.03 for offshorable) than low-skilled workers.

Thus, our results outline that the contractual form and the skill level are significant predictors of in-work poverty, whereas this is not the case for the offshorability of an occupational sector.

The variable ‘welfare regime’ in Model 5 (see Appendix Table 4) highlights significant cross-country differences. While welfare regimes seem unable to condition the effect of offshorability, which keeps its statistical insignificance, they exercise an autonomous effect on respondents’ likelihood of enduring in-work poverty. Notably, living in Southern Europe increases the probability of finding it difficult (+ 0.02) or very difficult (+ 0.01) to live on the present income compared to Eastern Europe. Patterns are reversed in Continental ( − 0.06 and  − 0.03) and Nordic countries ( − 0.07 and  − 0.03), where respondents are less likely to face in-work poverty than those residing in Eastern countries.

### Employment insecurity

The dependent variable ‘employment insecurity’ allows us to cast a light on whether respondents experienced unemployment in the last two years, and on the duration of their spells of unemployment. Average marginal effects from Model 2 (see Appendix Table 3) displayed in Fig. 4 Panel A show that being an atypical worker (versus being an insider) increases the predicted probabilities of having experienced an unemployment spell that lasted less than six months (+ 0.07), from six to twelve months (+ 0.08), or more than twelve months (+ 0.14). Not surprisingly, this pattern is even stronger for respondents who are currently unemployed, whose probability of having already spent more than twelve months without a job in the last two years is 0.56 points higher than that of insiders. Relevantly, and as for income insecurity, the solo self-employed’s exposure to employment insecurity is similar to that of atypical workers.

Here, too, the variable ‘offshorable’ does not reach statistical significance either alone or in interaction with respondents’ skill levels, suggesting that offshorability is not a good predictor of longer unemployment spells when unhampered by skill level.

Results from Model 2 confirm that skill level also has an autonomous effect on employment insecurity. According to Fig. 4 Panel B, irrespective of their employment sector’s exposure to international competition, highly skilled respondents are less likely to have been unemployed for more than twelve months ( − 0.05 for sheltered and  − 0.03 for offshorable) than their lower skilled colleagues.

Cross-country comparisons provide interesting insights. Notably, the likelihood of facing longer unemployment spells is higher in Southern countries (up to +0.07 for more than 12 months) and lower in Continental ones ( − 0.01) than it is in Eastern countries.

Here, too, no conditional effects are detected between welfare regime and offshorability in Model 6 (see Appendix Table 4), meaning that the risk of being unemployed is not higher for offshorable workers living in Continental, Nordic or Southern countries than for those living in Eastern countries. Moreover, if we look at the risk of unemployment by employment sector and skill level through cross-tabulations by country, only Finland complies with the expectations derived from the globalization losers’ literature: 47% of unskilled workers in offshorable sectors have been unemployed in the last two years, this share dropping to 42% for their counterparts in sheltered sectors. Conversely, 69% of highly skilled workers in offshorable sectors in Finland have never experienced unemployment, while this share drops to 62 per cent for their counterparts in sheltered sectors. In France, less skilled workers exposed to international competition are the most exposed to employment insecurity (38% experienced unemployment vs 34% for their counterparts in sheltered sectors), but the reverse pattern does not hold for their more skilled colleagues. Contrary to our initial expectations, in the remaining countries of our sample, low skilled workers are not more exposed to the risk of being unemployed when they work in offshorable sectors than when they work in sheltered ones.

### Access to social protection

Outsiders are more likely than their permanent counterparts to rely on social benefits different from pensions and/or old age benefits. The average marginal effects from Model 3 (see Appendix Table 3) displayed in Fig. 5 show that atypical workers are more likely than insiders to access social benefits (+ 0.08) and to declare that these benefits constitute the main or a very important part of their household income (+ 0.11). This result holds for the unemployed, but with a stronger magnitude, as the unemployed are 0.30 points more likely than insiders to declare that social benefits constitute an important part of their household income. It is very interesting to note that, on this front, the solo self-employed are quite different from outsiders, as they are only slightly more likely than insiders to declare that they receive social benefits and that such benefits constitute an important part of their household income (+ 0.03).

The fact that outsiders declared that they rely on social benefits more frequently than insiders does not mean, however, that their access to social protection systems has been equally easy across the surveyed countries. To start with, the average marginal effects for welfare regime in Model 7 (see Appendix Table 4) confirm the overall higher inclusiveness of the welfare state in Nordic countries compared to all other country groups (up to + 0.11). Moreover, cross-tabulations by country add that, on average, 45% of atypical workers had access to social benefits in Southern European countries (i.e. Greece, Italy and Spain) and 52% in Continental countries (i.e. France, Germany and the Netherlands), compared with 66% in universalistic Nordic countries (i.e. Sweden and Finland). This evidence is particularly striking if we consider that 75% of atypical workers declared having experienced a period of unemployment in the last two years in Southern European countries, whereas this share decreases to 64% in Nordic countries. In brief, atypical workers in Southern Europe are more likely to experience unemployment and less likely to have access to welfare benefits.

Moving to sectors’ exposure to international competition, the variable ‘offshorable’ does not reach conventional levels of statistical significance either alone or in interaction with respondents’ skill levels. Also, skill level does not perform well as a predictor of respondents’ likelihood of accessing social benefits.

Looking at cross-country variations, however, some interesting insights on the association between offshorability and access to social protection emerge. In Germany and Poland, less skilled workers in offshorable sectors seem to rely more heavily on social protection than those working in sheltered sectors: 36 per cent of them in Germany and 52% in Poland declare that they have benefitted from social benefits in the last two years, compared with 26% of their counterparts in sheltered sectors in Germany and 40% in Poland. The opposite is true in countries such as Greece (49% vs 57%), Italy (15% vs 19%), Hungary (24% vs 33%) and Spain (27% vs 33%), while in the other countries no significant differences emerge. Though more research is needed on this front—and to disentangle which social benefits these workers have access to—this evidence seems to suggest that workers exposed to international competition tend to access social benefits more easily in some countries than in others.

### Family dependency

The family can be important in reducing market insecurities by providing economic support, particularly in those countries where the welfare state is less inclusive and generous. Overall, our results support previous insights that familism is particularly relevant in Southern Europe (Ferrera 1996; Naldini 2003). The average marginal effects for welfare regime in Model 8 (see Appendix Table 4) show that the likelihood of relying on financial help from family is higher in Southern (up to + 0.04) and lower in Continental and Nordic countries (up to  − 0.03) than in Eastern ones. Cross-tabulations add that, on average, in Mediterranean countries, 32% of atypical workers and 45% of the unemployed sometimes or often ask for financial help from their families, shares significantly higher than in Continental (21% and 28%, respectively) and Northern European countries (31% and 25%, respectively).﻿

Also on this front, outsiders appear particularly exposed to the risk of depending on family help. The average marginal effects from Model 4 (see Appendix Table 3) displayed in Fig. 6 Panel A show that atypical workers are more likely than their permanent counterparts to rarely (+0.03), sometimes (+ 0.04) or often (+ 0.02) ask for financial help from family and close friends, while they are less likely than insiders never to ask ( − 0.10) for such help. Again, similarly to atypical workers but with a greater magnitude, the unemployed are more likely than insiders to rely financially on their families. On family dependency, the solo self-employed are similar to atypical workers, being more likely than insiders to ask for financial help from family or close friends (about + 0.04).

Neither working in an offshorable sector nor being low- or medium-skilled affects the likelihood of receiving financial help from family. In this regard, the only significant result is that highly skilled respondents, no matter whether working in sheltered or in offshorable sectors, tend never to ask for family help, unlike their lower skilled colleagues (Fig. 6 Panel B).

### Conclusion

Overall, Models 1–8 unanimously suggest that outsider status significantly and positively correlates with income and employment insecurities, as well as with the need to rely on financial help from the welfare system and the family. Working in occupational sectors exposed to international competition seems not to be a good predictor of these socio-economic risks. Not surprisingly, skill level seems to exercise a key role in sheltering individuals from socio-economic risks.

While the presence of conditional effects between the offshorability of the sector in which respondents work and welfare regimes is not supported, country-level characteristics autonomously affect the intensity of the socio-economic risks the active population has to face across EU countries. Notably, the risks of in-work poverty, longer unemployment spells and dependency on financial help from family are more salient in Southern countries than in Eastern ones, while Continental and Nordic countries seem overall to be better equipped to cope with these risks.

Overall, our results are in line with the literature outlining that the exposure of employment sectors to international competition is a much less important determinant of socio-economic risks and vulnerabilities than the individual position in the labour market (Iversen & Cusack 2000; Langsæther & Stubager 2019; Rehm 2009). The contractual form, rather than sectors’ exposure to globalization, plays the major role in triggering socio-economic insecurities and need for financial help. It is still possible, however, that similarly to so-called modernization losers—i.e. workers in routine jobs negatively affected by automation and technological change—low-skilled workers in offshorable jobs perceive a relative decline of status (Kurer 2020) and the potentiality of future vulnerabilities. Future research should address this hypothesis because, unfortunately, our data do not allow us to do it satisfactorily.^5^

## Discussion

This article has focused on the extent to which new labour market divides translate into individual-level socio-economic risks and vulnerabilities. We started our investigation by confirming that outsiders do not correspond to globalization losers, as these two segments of vulnerable workers do not overlap (Häusermann 2020). Rather, our analysis reveals that unemployment hits less skilled individuals harder, irrespective of the exposure of their employment sector to international competition, and that atypical employment affects slightly more workers in sheltered sectors than in offshorable sectors. It is important to emphasize, however, that this does not preclude the possibility that the processes of globalization and labour market deregulation are interrelated. Increasing global competition is indeed one of the usual suspects when trying to disentangle the causes of labour market deregulation in the European labour market, along with de-industrialization and technological change. Moreover, recent research has outlined how flexibilization also increases wage inequality among labour market insiders (Weisstanner 2021), and so it is possible that deregulation also had a negative impact on the living conditions of (dependent) globalization losers.

That said, outlining that outsiders and globalization losers are two different socio-economic groups paves the way for an important research question: are they exposed to similar socio-economic risks? Answering this question is crucial, as the living conditions individuals experience in their everyday lives contribute to shape their policy preferences and, consequently, their political behaviour.

Our main result is that the labour market position, rather than exposure to international competition (in combination or not with a low skill level), is a significant source of socio-economic risks. Labour market outsiders tend to have wages that do not allow their households to live decently, to be more frequently unemployed and for longer periods, to rely on social benefits—to which, however, they may have difficult or limited access—and, finally, to depend on financial support from family and/or close friends, when and as long as it is available. Overall, these results confirm that the insider–outsider divide is a crucial feature of European labour markets. Conversely, and to our surprise, working in sectors more exposed to international competition is not a source of socio-economic risks, irrespective of the dimension we look at. Indeed, workers in offshorable sectors do not face higher than average income and employment insecurities. That said, no matter the degree of offshorability of their employment sector, highly skilled workers tend to be more sheltered than their less skilled colleagues from income and employment insecurities and are less likely to rely on financial help from family and close friends, outlining the importance of education as a buffer against socio-economic insecurities in today’s post-industrial and globalized labour market.

Our findings also challenge the original formulation of the insider–outsider theory by outlining the existence of high heterogeneity within the outsiders’ world. Unemployment has a stronger negative effect on individual socio-economic conditions than atypical work. This is quite interesting because it seems to falsify one of the main arguments at the root of insider–outsider theory, according to which atypical workers and unemployed people should face the same difficulties and thus behave in the same way. Rather, they are differently exposed to socio-economic risks, and so they are likely to develop different redistributive preferences and, perhaps, express different party choices (Marx & Picot 2013; Negri 2019). Furthermore, this study reveals that the solo self-employed are closer to outsiders, especially to atypical workers, than to insiders in several dimensions of socio-economic risk, with one relevant exception: access to social protection. This signals the presence of (even) higher barriers to entrance to social protection systems for the solo self-employed compared to other outsiders rather than absence of need, as both their income and employment insecurities are similar to those of atypical workers. This evidence may contribute to explaining the puzzling electoral behaviour of the solo self-employed, and in particular their tendency to have more rightist positions on welfare policies than atypical workers and the unemployed (Jansen 2016; Negri 2019). On a more general note, these considerations suggest considering outsiderness as a sort of continuum, corresponding to different degrees of exposure to socio-economic risks (Emmenegger 2009; Marx & Picot 2013), with the solo self-employed at one extreme and the long-term unemployed at the other, passing through atypical workers.

Finally, this study confirms that there are more outsiders in the European periphery, particularly in Southern European countries, than in the core, and that in the European periphery labour market institutions and social protection systems tend to amplify rather than reduce socio-economic risks (Ferrera 1996; Schwander & Häusermann 2013). This result is relevant for at least two reasons: firstly, because, according to their intensity, new labour market divides may have had different political consequences in different European countries, contributing to explaining the emergence of varieties of populism (Caiani & Graziano 2019) and the different re-structuring of party systems in Europe (Hutter et al. 2018); secondly, and relatedly, because this ‘double dualization’ of Europe—the second referring to socio-economic vulnerabilities in peripheral and core countries drifting apart (Heidenreich 2016; Palier et al. 2018)—by increasing European countries’ socio-economic divergence, may have enduring consequences for the process of European integration and, in particular, for the political feasibility of a more social Europe (Ferrera 2017).

Therefore, this study, by investigating the exposure to socio-economic risks of different vulnerable segments of the labour market, has provided several relevant insights to enhance our theoretical and empirical knowledge about the individual-level causal mechanisms underlying the structuring of new political conflicts in Europe.

This paper has some limitations, though. Firstly, the analysis is based on cross-sectional survey data on ten countries, which makes it impossible to observe individuals’ working trajectories and to establish causal relationships. While for unemployed people we have information concerning their last occupational sector, this is not the case for atypical workers, for which we only know their current occupational sectors (see Sect. 4). Future research on longitudinal data may build on our results to check whether individuals trade more flexible contractual forms for more sheltered occupational sectors. Tabular associations in Table 1 seems to suggest this trade-off is not at play (atypical contracts are more frequent in sheltered sectors), but more fine-grained research is worthwhile.

Secondly, and relatedly, the 2019 REScEU Mass Survey covers only ten countries and data shortage does not allow us systematically to investigate cross-country differences, unless for descriptive tabular analyses. Additional research is thus needed to detect whether and how different types of welfare regime affect perceptions of socio-economic risks among vulnerable workers. Even more relevantly, a more in-depth investigation into how a country’s position in a given sectoral supply chain affects perceptions of insecurities among workers would be of the utmost importance.

Whilst acknowledging these limitations, this study has contributed to a clearer understanding of how new labour market divides generate socio-economic insecurities, a crucial condition for empirically grounded hypotheses on how new labour market inequalities transform political conflicts. Of note, the COVID-19 pandemic and the related economic crisis cannot but lay bare and exacerbate pre-existing labour market divides and gaps in social protection provisions. Our study provides sound evidence of the presence of diffused socio-economic risks unevenly affecting segments of workers throughout Europe. If not carefully addressed to ensure post-COVID-19 inclusive growth, such divides can only worsen, fostering unpredictable political reactions.

## Supplementary Information

Below is the link to the electronic supplementary material.

Supplementary file1 (PDF 323 kb)

## Data Availability

Data will be made available on the official website of the project SOLID – Policy Crisis and Crisis Politics, Sovereignty, Solidarity and Identity in the EU post 2008 (https://solid-erc.eu/).

## Footnotes

1 Several factors might contribute to different degrees of inclusiveness of needs-based social programmes, such as a very low-income threshold, strict behavioural requirements, the presence of administrative requirements discouraging potential applicants, a low take-up level, etc. (Eurofound, 2015).

2 The analysis is limited to the second wave of the REScEU Mass Survey as key variables for our analysis were not available in the first wave.

3 Every time a percentage is discussed in the main text, t-tests on the equality of means have been performed and are available upon request.

4 Low- and medium-skilled workers are contrasted with high-skilled ones as our sample covers major advanced European economies.

5 To partially address this point, we tested whether working in offshorable sectors and being an outsider in the labour market correlate with negative perceptions of the impact of immigration on domestic economy and of the EU integration process on job losses and social security. Results suggest that unemployed people and those working in offshorable sectors significantly blame immigration and the EU for their economic consequences. However, we are aware that our outcomes are poor proxies for the mechanism of fear of future economic deprivation outlined above. Indeed, they point to respondents’ policy preferences and thus are likely to be affected not only by respondents’ position in the labour market, but also by their ideological preferences. Estimates are available upon request.

## Code availability

Code will be made available on the official website of the project SOLID – Policy Crisis and Crisis Politics, Sovereignty, Solidarity and Identity in the EU post 2008 (https://solid-erc.eu/).
