# Supplementary material for: Disentangling (new) labour market divides: outsiders’ and globalization losers’ socio-economic risks in Europe
Source: Qual Quant. 2022 May 30;57(2):1561–85. doi: 10.1007/s11135-022-01414-9 (PMC9148940; doi:10.1007/s11135-022-01414-9)
Supplement: Supplementary file 1 — Supplementary file1 (PDF 323 kb) [file 11135_2022_1414_MOESM1_ESM.pdf]

Online Appendix

Appendix Table 1. Variables' description and operationalization

Variables' name	Operationalization	Corresponding items in the REScEU Mass Survey
Income insecurity	Ordinal variable equal to 1 if respondent lives comfortably on present income; 2 if s/he copes on present income; 3 if s/he finds it difficult on present income; 4 if s/he finds it very difficult on present income.	Q8_14
Employment insecurity	Ordinal variable equal to 1 if respondent has not experienced a continuous period of unemployment in the last two years; 2 if s/he has experienced a period of unemployment of less than 6 months; 3 if s/he has experienced a period of unemployment from 6 to 12 months; 4 if s/he has experienced a period of unemployment of more than 12 months.	Q8_7
Access to social protection	Ordinal variable equal to 1 if respondent has not received one or more types of social benefit (excluding old age/survivor pensions) in the last two years; 2 if s/he has received them but these benefits do not constitute his/her main or very important source of income; 3 if s/he has received them and they constitute his/her main or very important source of income.	Q8_8_1<br>Q8_8_2
Family dependency	Ordinal variable equal to 1 if respondent has never received financial help from close family or friends to pay bills, mortgage or rent, school fees or medical expenses; t 2 if s/he has rarely received this; 3 if s/he has sometimes received this; 4 if s/he has often received this.	Q8_15
Labour market status	Categorical variable that distinguishes among employers, solo self-employed, insiders, atypical workers and unemployed.	Q8_1<br>Q8_3
Offshorable (dummy)	Dummy variable equal to 1 if respondent works in an offshorable sector; 0 if s/he works in a sheltered sector.	Q8_2<br>Q8_4<br>Q8_5
Skill level	Categorical variable that distinguishes among low- (ISCED 2011 levels 0–2), medium- (levels 3–4) and high-skilled workers (levels 5–8).	sampling_levels_recode
Offshorable (categorical)	Categorical variable that distinguishes among 1. Low- and medium-skilled workers in offshorable sectors (globalization losers); 2. High-skilled workers in offshorable sectors (globalization winners); 3 low- and medium-skilled workers in sheltered sectors; and 4. High-skilled workers in sheltered sectors. It is used for Table 1.	Q8_2<br>Q8_4<br>Q8_5<br>sampling_levels_recode
Age	Ordinal variable that distinguishes among respondents aged 18–34, 35–54 or 55+.	Q0_2
Female	Dummy variable equal to 1 if respondent is female, 0 otherwise.	Q0_1
With partner	Dummy variable equal to 1 if respondent lives with a partner, 0 otherwise.	Q8_9
With children	Dummy variable equal to 1 if respondent lives with one or more children, 0 otherwise.	Q8_9
Urban area	Dummy variable equal to 1 if respondent lives in a town with more than 50,000 inhabitants, 0 otherwise.	Q8_17
Trade union member	Dummy variable equal to 1 if respondent is a trade union member, 0 otherwise.	Q6_9
Part-time worker	Dummy variable equal to 1 if respondent works less than 30 hours a week, 0 otherwise.	Q8_1
Welfare regime	Categorical variable that distinguishes among Southern (Greece, Italy and Spain), Eastern (Hungary and Poland - reference category), Continental (France, the Netherlands and Germany), and Nordic (Finland and Sweden) countries.	qcountry

Notes: For further details on question wording, please see the Codebook of the REScEU Mass Survey: https://resceu.eu/publications/working-papers/the-resceu-2019-mass-survey-codebook.html

Appendix Table 2. Descriptive statistics

**Variable**	**Mean**	**Std. Dev.**	**Min**	**Max**
Income insecurity	2.158	0.820	1	4
Employment insecurity	1.706	1.104	1	4
Access to social protection	0.499	0.740	0	2
Family dependency	1.677	0.937	1	4
Labour market status	3.187	0.767	1	5
Offshorable (dummy)	0.352	0.478	0	1
Skill level	2.279	0.681	1	3
Offshorable (categorical)	2.707	1.068	1	4
Age	1.927	0.680	1	3
Female	0.447	0.497	0	1
With partner	0.652	0.476	0	1
With children	0.474	0.499	0	1
Urban area	0.530	0.499	0	1
Trade union member	0.191	0.393	0	1
Part-time worker	0.135	0.342	0	1
Welfare regime	2.384	1.106	1	4

Appendix Table 3. Vulnerabilities of labour market outsiders and globalization losers (M1–M4 coefficients)

	**Income insecurity**	**Employment insecurity**	**Access to social protection**	**Family dependency**
	**M1**	**M2**	**M3**	**M4**
Employer	-0.315	0.440	0.145	0.479
	(0.150)**	(0.165)***	(0.175)	(0.153)***
Self-employed	0.587	0.797	0.265	0.560
	(0.085)***	(0.094)***	(0.098)***	(0.087)***
Atypical worker	0.309	1.369	0.895	0.459
	(0.066)***	(0.068)***	(0.071)***	(0.067)***
Unemployed	1.847	3.330	1.874	1.049
	(0.093)***	(0.105)***	(0.098)***	(0.094)***
Offshorable	-0.241	-0.172	0.059	0.175
	(0.133)*	(0.152)	(0.156)	(0.143)
Medium-skilled	-0.255	-0.419	-0.106	-0.084
	(0.087)***	(0.100)***	(0.101)	(0.094)
High-skilled	-0.824	-0.497	-0.177	-0.331
	(0.089)***	(0.101)***	(0.103)*	(0.096)***
Offsh*med.skilled	0.155	0.312	-0.132	-0.176
	(0.149)	(0.172)*	(0.174)	(0.160)
Offsh.*high.skilled	0.220	0.180	-0.165	-0.066
	(0.151)	(0.174)	(0.176)	(0.162)
Part-time	0.429	0.293	0.397	0.356
	(0.072)***	(0.079)***	(0.079)***	(0.075)***
Trade union	-0.029	-0.059	0.256	0.146
	(0.062)	(0.074)	(0.069)***	(0.067)**
34–54	0.353	-0.327	-0.219	-0.522
	(0.055)***	(0.062)***	(0.061)***	(0.056)***
55+	0.280	-0.442	-0.451	-1.269
	(0.067)***	(0.080)***	(0.079)***	(0.077)***
Female	0.165	0.090	0.039	0.168
	(0.046)***	(0.053)*	(0.052)	(0.049)***
With partner	-0.549	0.136	0.058	0.019
	(0.051)***	(0.060)**	(0.059)	(0.055)
With children	0.343	0.064	0.566	0.218
	(0.049)***	(0.058)	(0.057)***	(0.053)***
Urban area	0.003	0.103	-0.002	0.149
	(0.046)	(0.054)*	(0.052)	(0.049)***
Country dummies	*Yes*	*Yes*	*Yes*	*Yes*
Cut 1 – constant	-1.003	0.929	0.632	1.018
	(0.129)***	(0.149)***	(0.146)***	(0.144)***
Cut 2 – constant	1.659	1.676	1.912	2.105
	(0.131)***	(0.150)***	(0.148)***	(0.145)***
Cut 3 – constant	3.533	2.420		3.641
	(0.137)***	(0.152)***		(0.152)***
N	*7,633*	*7,495*	*7,379*	*7,501*
Log likelihood	*-8249.349*	*-6498.838*	*-6011.451*	*-7527.849*

Note: Ordered logistic regressions with country dummies. * $p < 0.1$; ** $p < 0.05$; *** $p < 0.01$

Appendix Table 4. Vulnerabilities of labour market outsiders and globalization losers (M5–M8 coefficients)

	**Income insecurity M5**	**Employment insecurity M6**	**Access to social protection M7**	**Family dependency M8**
Employer	-0.269*	0.458***	0.192	0.560***
	(0.149)	(0.164)	(0.174)	(0.151)
Self-employed	0.610***	0.805***	0.306***	0.652***
	(0.084)	(0.093)	(0.097)	(0.085)
Atypical worker	0.330***	1.373***	0.907***	0.497***
	(0.065)	(0.067)	(0.070)	(0.066)
Unemployed	1.850***	3.364***	1.897***	1.100***
	(0.092)	(0.104)	(0.096)	(0.093)
Offshorable	-0.091	0.103	-0.083	0.067
	(0.097)	(0.121)	(0.118)	(0.105)
Medium-skilled	0.015	-0.256***	0.071	0.102
	(0.070)	(0.081)	(0.082)	(0.076)
High-skilled	-0.548***	-0.352***	-0.003	-0.095
	(0.072)	(0.083)	(0.083)	(0.078)
Part-time	0.348***	0.251***	0.337***	0.325***
	(0.072)	(0.078)	(0.078)	(0.075)
Trade union	-0.103*	-0.098	0.193***	0.081
	(0.061)	(0.074)	(0.068)	(0.067)
34–54	0.310***	-0.323***	-0.223***	-0.517***
	(0.054)	(0.062)	(0.061)	(0.055)
55+	0.178***	-0.465***	-0.509***	-1.316***
	(0.067)	(0.079)	(0.078)	(0.076)
Female	0.159***	0.089*	0.051	0.154***
	(0.045)	(0.053)	(0.051)	(0.048)
With partner	-0.561***	0.110*	0.033	-0.002
	(0.051)	(0.060)	(0.058)	(0.054)
With children	0.345***	0.088	0.572***	0.222***
	(0.048)	(0.058)	(0.055)	(0.052)
Urban area	-0.013	0.092*	0.003	0.171***
	(0.045)	(0.053)	(0.051)	(0.048)
South	0.192**	0.757***	0.031	0.410***
	(0.079)	(0.094)	(0.092)	(0.083)
Continental	-0.471***	-0.238**	0.045	-0.348***
	(0.080)	(0.102)	(0.093)	(0.088)
Nordic	-0.576***	0.296***	0.843***	-0.240**
	(0.090)	(0.108)	(0.100)	(0.098)
Offsh*South	-0.036	-0.190	-0.235	-0.049
	(0.128)	(0.151)	(0.153)	(0.134)
Offsh*Cont.	-0.059	-0.050	0.191	-0.051
	(0.130)	(0.165)	(0.153)	(0.143)
Offsh*Nordic	0.029	-0.030	-0.053	-0.039
	(0.145)	(0.173)	(0.161)	(0.156)
Cut 1 – constant	-1.707***	1.209***	1.323***	0.419***
	(0.109)	(0.127)	(0.126)	(0.114)
Cut 2 – constant	0.871***	1.949***	2.577***	1.465***
	(0.107)	(0.128)	(0.129)	(0.116)
Cut 3 – constant	2.701***	2.687***		2.953***
	(0.115)	(0.131)		(0.123)
N	*7633*	*7495*	*7379*	*7501*
Log likelihood	*-8415.295*	*-6540.76*	*-6111.033*	*-7684.306*

Note: Ordered logistic regressions with 'welfare regime'. * $p < 0.1$; ** $p < 0.05$; *** $p < 0.01$
